# RETRACTED: Dahal et al. PERK Is Critical for Alphavirus Nonstructural Protein Translation. *Viruses* 2021, *13*, 892

**DOI:** 10.3390/v17030318

**Published:** 2025-02-26

**Authors:** Bibha Dahal, Caitlin W. Lehman, Ivan Akhrymuk, Nicole R. Bracci, Lauren Panny, Michael D. Barrera, Nishank Bhalla, Jonathan L. Jacobs, Jonathan D. Dinman, Kylene Kehn-Hall

**Affiliations:** 1 National Center for Biodefense and Infectious Diseases, School of Systems Biology, George Mason University, Manassas, VA 20110, USA; bdahal@gmu.edu (B.D.); Woodsonc@vt.edu (C.W.L.); Iakhrymu@vt.edu (I.A.); nbracci@vt.edu (N.R.B.); laurenpanny@vt.edu (L.P.); mbarrer@gmu.edu (M.D.B.); nbhalla@gmu.edu (N.B.); 2 Department of Biomedical Sciences and Pathobiology, Virginia-Maryland College of Veterinary Medicine, Virginia Polytechnic Institute and State University, Blacksburg, VA 24061, USA; 3 American Type Culture Collection, Manassas, VA 20110, USA; jjacobs@atcc.org; 4 Department of Cell Biology and Molecular Genetics, University of Maryland, College Park, MD 20742, USA; dinman@umd.edu; 5 Center for Zoonotic and Arthropod-Borne Pathogens, Virginia Polytechnic Institute and State University, Blacksburg, VA 24061, USA

The journal *Viruses* retracts the article “PERK Is Critical for Alphavirus Nonstructural Protein Translation” [1] cited above.

Following publication, the authors contacted the Editorial Office regarding reproducibility issues relating to reagent contamination [1].

Adhering to our standard procedure, the Editorial Board reviewed the supplementary information provided, including raw material relating to the authors’ attempts to replicate the results, and agreed with the authors that the central findings of this study could not be sustained and can no longer be considered valid. As a result, the Editorial Board and the authors have decided to retract this paper as per MDPI’s retraction policy (https://www.mdpi.com/ethics#_bookmark30).

This retraction was approved by the Editor-in-Chief of the journal *Viruses*.

The authors agreed to this retraction.
